# Interference-exact radiative transfer equation

**DOI:** 10.1038/s41598-017-11753-5

**Published:** 2017-09-14

**Authors:** Mikko Partanen, Teppo Häyrynen, Jani Oksanen

**Affiliations:** 1 Engineered Nanosystems group, School of Science, Aalto University, P.O. Box 12200, 00076 Aalto, Finland; 2 DTU Fotonik, Department of Photonics Engineering, Technical University of Denmark, Ørsteds Plads, Building 343, DK-2800 Kongens Lyngby, Denmark

## Abstract

The Purcell effect, i.e., the modification of the spontaneous emission rate by optical interference, profoundly affects the light-matter coupling in optical resonators. Fully describing the optical absorption, emission, and interference of light hence conventionally requires combining the full Maxwell’s equations with stochastic or quantum optical source terms accounting for the quantum nature of light. We show that both the nonlocal wave and local particle features associated with interference and emission of propagating fields in stratified geometries can be fully captured by local damping and scattering coefficients derived from the recently introduced quantized fluctuational electrodynamics (QFED) framework. In addition to describing the nonlocal optical interference processes as local directionally resolved effects, this allows reformulating the well known and widely used radiative transfer equation (RTE) as a physically transparent interference-exact model that extends the useful range of computationally efficient and quantum optically accurate interference-aware optical models from simple structures to full optical devices.

## Introduction

The radiative transfer equation (RTE) is a commonly used model to describe absorption, emission, and scattering processes of light propagating through turbid macroscopic media^1–4^. The well known main limitation of the classical RTE model is that it does not account for the full range of interference effects present in nonuniform media^2, 5^. This limitation fundamentally arises from the challenge to separately attribute the interference induced modifications in the light-matter coupling^6–8^ to the propagating modes of the system. To overcome this limitation and to provide a physically transparent relation between the various optical density of states concepts^7, 9–11^ and the local propagating field interactions, we derive quantum optically exact damping and scattering coefficients that allow including all interference related effects directly in the RTE model of stratified media. The derivation requires the ability to unambiguously separate the propagating optical fields into left- and right-propagating components which has only recently become possible with the introduction of the QFED framework^9, 12–15^. The QFED framework unambiguously combines the quantized Maxwell’s equations and the related quantum optical source terms^16–18^ with the canonical commutation relations of the ladder operators of the fields, thereby allowing, e.g., to identify the propagating field photon numbers^14^ and to study the formation of thermal balance in resonator structures using the concept of photon number^12^. Here, the connection between the QFED and RTE makes the essential initial step towards converting RTE into a scalable and all-inclusive optical model with interference modulated model parameters and transparent physical interpretation. It also allows extending many quantum models^19–22^ to account for interference.

## RTE model

The connection between the QFED and the RTE model can be established by comparing pointwise the rate of change of the photon number due to the absorption and emission in both the RTE and QFED models. The comparison is started by first calculating the derivative of the left (−) and right (+) propagating field photon-number expectation values of the QFED method along the *z*-axis for angular frequency *ω* and polarization *σ* ∈ {TE, TM}, as presented in Methods in Eq. (5). The expectation value here corresponds to the average occupation number of the pertinent optical modes, fully preserving the nonlocal features of the field. Then the propagating photon numbers and their derivatives are substituted in the RTE model of stratified media written as

1 $$\begin{array}{rcl}\frac{d}{dz}\langle {\hat{n}}_{\pm ,\sigma }(z,K,\omega )\rangle  & = & \mp {\alpha }_{\pm ,\sigma }(z,K,\omega )[\langle {\hat{n}}_{\pm ,\sigma }(z,K,\omega )\rangle -\langle {\hat{\eta }}_{\sigma }(z,K,\omega )\rangle ]\\  & = & \pm {\beta }_{\pm ,\sigma }(z,K,\omega )[\langle {\hat{n}}_{\mp ,\sigma }(z,K,\omega )\rangle -\langle {\hat{\eta }}_{\sigma }(z,K,\omega )\rangle \mathrm{].}\end{array}$$

Here *K* is the wave vector component in the *x*−*y* plane and we have allowed a general position- and direction-dependent form for the damping coefficients *α*_±,*σ*_(*z*, *K*, *ω*) and the scattering coefficients *β*_±,*σ*_(*z*, *K*, *ω*). The full derivation of these coefficients is represented in Methods, where they are shown to be given by

2 $$\begin{array}{rcl}{\alpha }_{\pm ,\sigma }(z,K,\omega ) & = & \begin{array}{c}\frac{1}{2{\rho }_{\sigma }{\rho }_{{\rm{IF}},\sigma }}[\frac{\partial {\rho }_{\sigma }}{\partial z}{\rho }_{{\rm{NL}}\pm ,\sigma }-{\rho }_{\sigma }\frac{\partial {\rho }_{{\rm{NL}}\pm ,\sigma }}{\partial z}-{\rho }_{{\rm{NL}}\mp ,\sigma }{\int }_{{z}^{-}}^{{z}^{+}}\frac{\partial {\rho }_{{\rm{NL}}\pm ,\sigma }}{\partial z}dz^{\prime} ],\end{array}\\ {\beta }_{\pm ,\sigma }(z,K,\omega ) & = & \frac{1}{2{\rho }_{\sigma }{\rho }_{{\rm{IF}},\sigma }}[\frac{\partial {\rho }_{\sigma }}{\partial z}{\rho }_{{\rm{NL}}\pm ,\sigma }-{\rho }_{\sigma }\frac{\partial {\rho }_{{\rm{NL}}\pm ,\sigma }}{\partial z}-{\rho }_{{\rm{NL}}\pm ,\sigma }{\int }_{{z}^{-}}^{{z}^{+}}\frac{\partial {\rho }_{{\rm{NL}}\pm ,\sigma }}{\partial z}dz^{\prime} ],\end{array}$$

where the terms *ρ*_*σ*_ and *ρ*_*i*,*σ*_, *i* ∈ {IF, NL±} are the local and nonlocal densities of states presented in Supplemental Material and in ref. 9.

## Results

### Homogeneous medium

In general, the damping and scattering coefficients in Eq. (2) can be position dependent. This is naturally not the case in a homogeneous space, where the damping and scattering coefficients are constant and separately equal for fields propagating in different directions, i.e., *α*_+,*σ*_(*z*, *K*, *ω*) = *α*_−,*σ*_(*z*, *K*, *ω*) = *α*_±,*σ*_ and *β*_+,*σ*_(*z*, *K*, *ω*) = *β*_−,*σ*_(*z*, *K*, *ω*) = *β*_±,*σ*_. As shown in the Supplemental Material, substituting the densities of states corresponding to the homogeneous space Green’s functions into the damping and scattering coefficients in Eq. (2) leads to damping and scattering coefficients

3 $${\alpha }_{\pm ,\sigma }={k}_{z,{\rm{i}}}({\psi }_{\sigma }+{\psi }_{\sigma }^{-1}),\quad {\beta }_{\pm ,\sigma }={k}_{z,{\rm{i}}}({\psi }_{\sigma }-{\psi }_{\sigma }^{-1}),$$

where *k*_*z*,*i*_ is the imaginary part of the wave vector *z* component ${k}_{z}=\sqrt{{k}^{2}-{K}^{2}}$, the wavenumber is *k* =* nω*/*c*, *n* is the refractive index, *c* is the speed of light in vacuum, and the parameter *ψ*_σ_ is given for the TE and TM polarizations by

4 $${\psi }_{{\rm{TE}}}=\frac{|k{|}^{2}+|{k}_{z}{|}^{2}+{K}^{2}}{\mathrm{2|}\mu |{k}_{{\rm{r}}}{\rm{Re}}({k}_{z}/\mu )},\quad {\psi }_{{\rm{TM}}}=\frac{|k{|}^{2}+|{k}_{z}{|}^{2}+{K}^{2}}{\mathrm{2|}\varepsilon |{k}_{{\rm{r}}}{\rm{Re}}({k}_{z}/\varepsilon )}\mathrm{.}$$

Here *k*_*r*_ is the real part of the wavenumber and *ε* and *μ* are the relative permittivity and permeability of the medium, which are related to the refractive index as $n=\sqrt{\varepsilon \mu }$.

In a lossless uniform medium, the damping and scattering coefficients are all zero for propagating modes as the imaginary part of the *z*-component of the wave vector is zero. In homogeneous lossy media, on the other hand, the damping and scattering coefficients are both positive and the damping coefficients *α*_±,*σ*_ are larger than the scattering coefficients *β*_±,*σ*_. For normal incidence with *K* = 0, the coefficients for the TE and TM polarizations are equal as expected. In a purely dielectric medium with $n=\sqrt{\varepsilon }$, the damping and scattering coefficients in Eq. (3) simplify for *K* = 0 to ${\alpha }_{\pm }={k}_{{\rm{i}}}(|n{|}^{2}/{n}_{{\rm{r}}}^{2}+{n}_{{\rm{r}}}^{2}/|n{|}^{2})$ and ${\beta }_{\pm }={k}_{{\rm{i}}}(|n{|}^{2}/{n}_{{\rm{r}}}^{2}-{n}_{{\rm{r}}}^{2}/|n{|}^{2})$ where *k*_i_ is the imaginary part of the wavenumber and *n*_r_ is the real part of the refractive index. In the limit of small losses, one can approximate |*n*| ≈ *n*_r_ and the damping and scattering coefficients become approximately equal to the classical expressions *α*_±_ ≈ 2*k*_i_ and *β*_±_ ≈ 0. For larger losses, however, the scattering coefficients become nonzero. For example, in the case of a dielectric material with refractive index *n* = 2 + *i*, the coefficients are given by *α*_±_ = 2.05*k*_0_ and *β*_±_ = 0.45*k*_0_, where *k*_0_ = *ω*/*c* is the wavenumber in vacuum. The damping coefficient is still close to the classical value, but the scattering coefficient clearly deviates from the classical result of zero, indicating that a part of the photons is scattered backwards due to the induced electric and magnetic dipoles in the medium.

### Single-interface geometry

**Figure 1:**
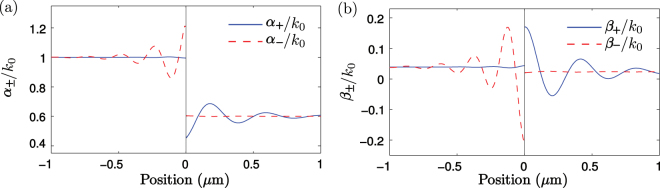
(**a**) Damping coefficients *α*_+_ and *α*_−_ and (**b**) scattering coefficients *β*_+_ and *β*_−_ in units of *k*_0_ for photon energy *ħω* = 1 eV (*λ* = 1.24 *μ*m) as a function of position in the vicinity of an interface between two lossy dielectric media. The medium on the left has a refractive index $\sqrt{{\varepsilon }_{1}}=2.5+0.5i$ and the medium on the right has a refractive index $\sqrt{{\varepsilon }_{2}}=1.5+0.3i$. The vertical solid line denotes the interface between the media.

To illustrate the general position dependence of the damping and scattering coefficients, we next study the damping and scattering coefficients for photon energy *ħω* = 1 eV (*λ* = 1.24 *μ*m) in the vicinity of an interface between two lossy media with refractive indices ${n}_{1}=\sqrt{{\varepsilon }_{1}}=2.5+0.5i$ and ${n}_{2}=\sqrt{{\varepsilon }_{2}}=1.5+0.3i$. The normal incidence damping and scattering coefficients for the single interface structure are given in Supplemental Material and, in Fig. 1, they are plotted as a function of the position. In Fig. 1(a), the damping coefficients *α*_+_ and *α*_−_ reach homogeneous field values 1.00077*k*_0_ and 0.60046*k*_0_ far from the interface, whereas, near the interface, they oscillate. The above homogeneous field values of the damping coefficients are seen to be very close to the classical values 2*k*_1,*i*_ = *k*_0_ and 2*k*_2,*i*_ = 0.6*k*_0_. The oscillations of the damping coefficients near the interface originate from the interference and the modified position-dependent emission and absorption rates in analogy with the Purcell effect^6^. The oscillations in both the scattering and damping coefficients are substantially larger in the propagation directions away from the interface, suggesting that the photons propagating away from the interface experience stronger interference effects. For certain material combinations, the oscillations in the damping coefficients can become much larger and it is also possible for the damping coefficients to obtain negative values suggesting that the field may experience local amplification.

The scattering coefficients *β*_+_ and *β*_−_ in Fig. 1(b) have significantly smaller values than the damping coefficients *α*_+_ and *α*_−_ in Fig. 1(a). This is expected as the change of the field propagating in one direction generally depends more on the field itself than on the field propagating in the other direction. In addition, also the scattering coefficients *β*_+_ and *β*_−_ can obtain negative values near the interfaces due to interference. On the left and right, the oscillations in the scattering coefficients die out and saturate to the homogeneous space values 0.03923*k*_0_ and 0.02354*k*_0_, which are nonzero, thus slightly deviating from the classical results.

### Two-interface resonator

Next we study the damping and scattering coefficients in a two-interface resonator formed by a dielectric slab with a refractive index $n=\sqrt{\varepsilon }=2+0.1i$ placed in vacuum. We also compare the results of our interference-exact RTE model and the classical field-based methods directly solving Maxwell’s equations with appropriate boundary conditions. For a concise comparison, we use the negative divergence of the Poynting vector −∇ · **S** (here **S** is calculated using Eq. (6) in ref. 14), which describes the net absorption rate that is well-known to oscillate inside lossy resonant structures due to interference and the related Purcell effect^6^. These oscillations cannot be described correctly by using models such as the conventional RTE model which neglects essentially all interference effects, e.g., coherent backscattering^5^.

**Figure 2:**
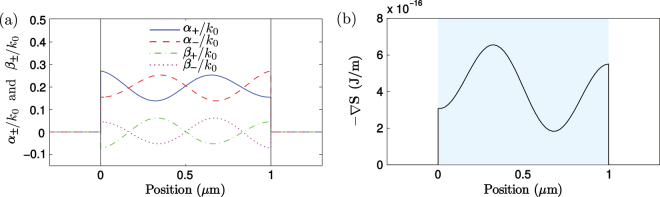
(**a**) The damping coefficients *α*_+_ and *α*_−_ and scattering coefficients *β*_+_ and *β*_−_ in units of *k*_0_ and (**b**) the spectral net absorption rate as a function of position for normal incidence in the geometry of a dielectric slab in vacuum. The slab medium has a refractive index $\sqrt{\varepsilon }=2+0.1i$. The assumed photon energy *ħω* = 0.46 eV (*λ* = 2.68 *μ*m) corresponds to the second resonance of the reflected field. The vertical solid lines denote the slab boundaries. The total power reflection coefficient of the slab is *R* = 0.26.

Figure 2(a) shows the damping and scattering coefficients as a function of position for photon energy *ħω* = 0.46 eV (*λ* = 2.68 *μ*m) and for normal incidence in the vicinity of the dielectric slab. The used photon energy corresponds to the second constructive interference of the field reflected from the slab, i.e., the intensity of the reflected field obtains its second maximum when it is plotted as a function of photon energy. One can clearly see that the damping and scattering coefficients are oscillating in the slab. Outside the slab, the coefficients are zero as there are no losses in vacuum.

When comparing the results of the derived interference-exact RTE model in Fig. 2(b) with the results of the classical solution of Maxwell’s equations, we assume normal incidence, set the source-field temperature of the resonator to zero as *T* = 0 K, and use the initial condition that, on the right, there is only a right propagating field with a fixed average photon number, i.e., $\langle {\hat{n}}_{+,\sigma }(z,K,\omega )\rangle =1$ and $\langle {\hat{n}}_{-,\sigma }(z,K,\omega )\rangle =0$. In the classical method, this corresponds to fixing the electric and magnetic fields on the right such that the resulting Poynting vector is the same as that in our interference-exact RTE model. The net absorption rates calculated by using the interference-exact RTE model and the classical method in Fig. 2(b) are equal within the precision of the numerical accuracy of the computations. This clearly demonstrates that by using the derived position-dependent damping and scattering coefficients, the conventional RTE model can be extended to account for all interference effects. More extensive comparisons between the interference-exact RTE model and full FED based calculations have additionally indicated that the results are also generally valid for other angles of incidence and resonator source fields *T* > 0 K.

## Conclusions

In conclusion, we have used the newly developed QFED framework to derive the interference modified local field-matter coupling strengths of propagating fields and to extend the widely deployed RTE model so that it receives the ability to also fully capture interference effects in stratified geometries. The approach involves deriving the quantum optically exact damping and scattering coefficients from the position dependent expectation values of the propagating photon-number operators provided by the QFED framework. This approach allows providing an accurate and transparent local physical picture of interference as a mechanism that modulates the strength of the light-matter interactions. In addition to the physical transparency, the approach is expected to be very useful from the computational point of view when the Green’s functions are known analytically or can be easily solved for, as the photon numbers no longer exhibit the strong oscillations throughout the simulation space, as is the case for the electric fields in conventional Maxwell’s equation based models. This allows substantially relaxing the requirements set on the problem discretization away from interfaces. Furthermore, the model is also expected to enable new possibilities for modeling quantum effects (like coherence and collective effects) of the fields in macroscopic structures or devices. Overall, the presented interference exact RTE model therefore allows substantial widening of the use of RTE-based models to a wide variety of new geometries involving e.g. thin-films and resonators which have not been previously accessible to the simple RTE-based methods.

## Methods

### Number of photons and densities of states

In contrast to other approaches used to describe fields in lossy resonant media, the fundamental requirement of the QFED is the preservation of the canonical commutation relation $[\hat{a}(z,\omega ),{\hat{a}}^{\dagger }(z,\omega ^{\prime} )]=\delta (\omega -\omega ^{\prime} )$ of photon ladder operators at position *z* for angular frequency *ω* also in resonant structures^12^. This requirement leads to conceptually simple definitions for the position-dependent photon-number and ladder operators as weighted sums over the incident fields and the noise. As demonstrated previously in ref. 14 for the left (−) and right (+) propagating fields, the position-dependent expectation values $\langle {\hat{n}}_{\pm ,\sigma }(z,\omega )\rangle$ of the photon-number operators ${\hat{n}}_{\pm ,\sigma }(z,\omega )$ for the mode with angular frequency *ω* and polarization *σ* ∈ {TE, TM} read as^14^

5 $$\langle {\hat{n}}_{\pm ,\sigma }(z,K,\omega )\rangle =\frac{1}{{\rho }_{\sigma }(z,K,\omega )}{\int }_{-\infty }^{\infty }{\rho }_{{\rm{NL}}\pm ,{\rm{\sigma }}}(z,K,\omega ,z^{\prime} )\langle {\hat{\eta }}_{\sigma }(z^{\prime} ,K,\omega )\rangle dz^{\prime} \mathrm{.}$$

Here *K* is the wave vector component in the *x*−*y* plane, *ρ*_*σ*_(*z*, *K*, *ω*) is the local density of states (LDOS)^9, 14, 15^ and we refer to the weighting coefficients *ρ*_NL±,*σ*_(*z*, *K*, *ω*, *z*′) as the nonlocal densities of states (NLDOSs) of the left and right propagating fields. These NLDOSs are given as sums and differences of the NLDOSs of the total electromagnetic field *ρ*_NL,*σ*_(*z*, *K*, *ω*, *z*′) and the interference densities of states (IFDOSs) *ρ*_IF,*σ*_(*z*, *K*, *ω*, *z*′) as *ρ*_NL±,*σ*_(*z*, *K*, *ω*, *z*′) = *ρ*_NL,*σ*_(*z*, *K*, *ω*, *z*′) ± *ρ*_IF,*σ*_(*z*, *K*, *ω*, *z*′)^14^, which are all related to the electromagnetic Green’s functions of the system. These densities of states have been originally derived in ref. 14 and 9 and, in Supplemental Material, they are explicitly given in terms of the spectral dyadic Green’s function components for stratified media. In Eq. (5), $\langle {\hat{\eta }}_{\sigma }(z,K,\omega )\rangle$ is the source-field photon-number expectation value given for thermal fields by the Bose-Einstein distribution $\langle {\hat{\eta }}_{\sigma }(z,K,\omega )\rangle =\mathrm{1/[}{e}^{\hslash \omega /({k}_{{\rm{B}}}T(z))}-\mathrm{1]}$, where *T*(*z*) is the position-dependent temperature profile of the medium.

### Derivation of the RTE coefficients

Substituting the integral expressions for the photon-number expectation values of the QFED framework as given by Eq. (5) into the RTE model in Eq. (1) and omitting the arguments *z*, *z*′, *K*, and *ω* for brevity, we obtain

6 $$\begin{array}{rcl}{\int }_{-\infty }^{\infty }[\frac{1}{{\rho }_{\sigma }}\frac{\partial {\rho }_{{\rm{NL}}\pm ,\sigma }}{\partial z}-\frac{1}{{\rho }_{\sigma }^{2}}\frac{\partial {\rho }_{\sigma }}{\partial z}{\rho }_{{\rm{NL}}\pm ,\sigma }]\langle {\hat{\eta }}_{\sigma }\rangle dz^{\prime}  & = & {\int }_{-\infty }^{\infty }[\mp {\alpha }_{\pm ,\sigma }(\frac{{\rho }_{{\rm{NL}}\pm ,\sigma }}{{\rho }_{\sigma }}-\delta (z-z^{\prime} ))\\  &  & \pm {\beta }_{\pm ,\sigma }(\frac{{\rho }_{{\rm{NL}}\mp ,\sigma }}{{\rho }_{\sigma }}-\delta (z-z^{\prime} ))]\langle {\hat{\eta }}_{\sigma }\rangle dz^{\prime} ,\end{array}$$

where the source-field terms $\langle {\hat{\eta }}_{\sigma }\rangle$ of Eq. (1) have been imported within the integrals by using suitable *δ*-function representations. To determine the RTE-coefficients *﻿α*_±,σ﻿_and *β*_±,σ﻿_, we require that the integrands on the left and right side of Eq. (6) must be equal at all positions *z* and *z*′, which corresponds to requiring that the RTE model is valid for arbitrary material temperature distributions represented by the position-dependent source-field photon-number expectation value $\langle {\hat{\eta }}_{\sigma }\rangle$. Although it is not immediately apparent, Eq. (6) only provides two linearly independent components, one for the case *z* = *z*′ and one for any *z* ≠ *z*′. This enables us to separate Eq. (6) into an equation group of two linearly independent equations. For the case *z* = *z*′ we obtain an equation

7 $$\frac{1}{{\rho }_{\sigma }}{\int }_{{z}^{-}}^{{z}^{+}}\frac{{\rm{\partial }}{\rho }_{{\rm{N}}{\rm{L}}\pm ,\sigma }}{{\rm{\partial }}z}d{z}^{{\rm{^{\prime} }}}=\pm {\alpha }_{\pm ,\sigma }\mp {\beta }_{\pm ,\sigma },$$

where *z* ^−^ and *z* ^+^ denote values on the left and right infinitesimally close to *z*. For *z* ≠ *z*′, by setting the integrands on the left and right side of Eq. (6) equal, we respectively obtain an equation

8 $$\frac{\partial {\rho }_{{\rm{NL}}\pm ,\sigma }}{\partial z}-\frac{1}{{\rho }_{\sigma }}\frac{\partial {\rho }_{\sigma }}{\partial z}{\rho }_{{\rm{NL}}\pm ,\sigma }=\mp {\alpha }_{\pm ,\sigma }{\rho }_{{\rm{NL}}\pm ,\sigma }\pm {\beta }_{\pm ,\sigma }{\rho }_{{\rm{NL}}\mp ,\sigma }\mathrm{.}$$

Solving the pair of equations formed by Eqs (7) and (8) results in the damping and scattering coefficients in Eq. (2) which provide a complete and quantum optically accurate description of how the light-matter interaction strength is modified by interference.

### Solution of the RTE model

To use the damping and scattering parameters in writing the solution of the RTE model in a general form, again omitting the function arguments *K* and *ω* for brevity, the RTE model in Eq. (1) can be written as a matrix equation

9 $$\frac{d}{dz}{{\bf{n}}}_{\sigma }(z)=-{{\boldsymbol{\alpha }}}_{\sigma }(z)[{{\bf{n}}}_{\sigma }(z)-{{\boldsymbol{\eta }}}_{\sigma }(z)],$$

where **n**_*σ*_(*z*) is a vector formed from the photon numbers of the fields propagating in different directions as ${{\bf{n}}}_{\sigma }(z)={[\langle {\hat{n}}_{+,\sigma }(z)\rangle ,\langle {\hat{n}}_{-,\sigma }(z)\rangle ]}^{T}$, ***η***_*σ*_(*z*) is a corresponding vector for the source field given by ${{\boldsymbol{\eta }}}_{\sigma }(z)={[\langle {\hat{\eta }}_{\sigma }(z)\rangle ,\langle {\hat{\eta }}_{\sigma }(z)\rangle ]}^{T}$, and

10 $${{\boldsymbol{\alpha }}}_{\sigma }(z)=[\begin{array}{cc}{\alpha }_{+,\sigma } & -{\beta }_{+,\sigma }\\ {\beta }_{-,\sigma } & -{\alpha }_{-,\sigma }\end{array}].$$

With the boundary condition **n**_*σ*_(*z*_0_) = **n**_*σ*,0_ the solution to Eq. (9) is then given by

11 $${{\bf{n}}}_{\sigma }(z)={e}^{-{\int }_{{z}_{0}}^{z}{{\boldsymbol{\alpha }}}_{\sigma }({z}^{{\rm{^{\prime} }}})d{z}^{{\rm{^{\prime} }}}}{{\bf{n}}}_{\sigma ,0}+{\int }_{{z}_{0}}^{z}{e}^{-{\int }_{{z}^{{\rm{^{\prime} }}}}^{z}{{\boldsymbol{\alpha }}}_{\sigma }({z}^{{\rm{^{\prime} }}{\rm{^{\prime} }}})d{z}^{{\rm{^{\prime} }}{\rm{^{\prime} }}}}{{\boldsymbol{\alpha }}}_{\sigma }({z}^{{\rm{^{\prime} }}}){{\boldsymbol{\eta }}}_{\sigma }({z}^{{\rm{^{\prime} }}})d{z}^{{\rm{^{\prime} }}}.$$

Equation (11) is fully analogous with the conventional solution of the RTE model with the conventional damping coefficient replaced by the matrix ***α***_*σ*_(*z*). In contrast, the equation is dramatically different from similar direct solutions of both the homogeneous Maxwell’s equations or the classical FED, both requiring very dense calculation grids in numerical computations and latter being also stochastic in nature. Furthermore, the form of Eq. (11) is equivalent with the conventional quantum optical input-output approach^19^, which also uses the conventional damping coefficient in the place of the present matrix presentation ***α***_*σ*_(*z*).

## Electronic supplementary material

Supplementary Information

## References

[1] Chandrasekhar, S. *Radiative transfer* (Dover, New York, 1960).

[2] Mishchenko MI (2014). Electromagnetic Scattering by Particles and Particle Groups: An Introduction.

[3] Mishchenko MI (2014). Directional radiometry and radiative transfer: The convoluted path from centuries-old phenomenology to physical optics. J. Quant. Spectrosc. Radiat. Transfer.

[4] Mishchenko MI (2006). Maxwell’s equations, radiative transfer, and coherent backscattering: A general perspective. J. Quant. Spectrosc. Radiat. Transfer.

[5] Mishchenko MI, Travis LD, Lacis AA (2006). Multiple Scattering of Light by Particles: Radiative Transfer and Coherent Backscattering.

[6] Purcell, E. M. Spontaneous emission probabilities at radio frequencies. *Phys. Rev*. **69**, 681 (1946). Note B10 in “Proceedings of the American Physical Society” *Phys. Rev*. **69**, 674, doi:10.1103/PhysRev.69.674.2 (1946).

[7] Ginzburg P (2017). Spontaneous emission in non-local materials. Light Sci. Appl..

[8] Poddubny AN, Belov PA, Ginzburg P, Zayats AV, Kivshar YS (2012). Microscopic model of Purcell enhancement in hyperbolic metamaterials. Phys. Rev. B.

[9] Partanen M, Häyrynen T, Tulkki J, Oksanen J (2017). Quantized fluctuational electrodynamics for three-dimensional plasmonic structures. Phys. Rev. A.

[10] Joulain K, Carminati R, Mulet J-P, Greffet J-J (2003). Definition and measurement of the local density of electromagnetic states close to an interface. Phys. Rev. B.

[11] Narayanaswamy A, Chen G (2010). Dyadic green’s functions and electromagnetic local density of states. J. Quant. Spectrosc. Radiat. Transfer.

[12] Partanen M, Häyrynen T, Oksanen J, Tulkki J (2014). Thermal balance and photon-number quantization in layered structures. Phys. Rev. A.

[13] Partanen M, Häyrynen T, Oksanen J, Tulkki J (2014). Unified position-dependent photon-number quantization in layered structures. Phys. Rev. A.

[14] Partanen M, Häyrynen T, Tulkki J, Oksanen J (2015). Commutation-relation-preserving ladder operators for propagating optical fields in nonuniform lossy media. Phys. Rev. A.

[15] Partanen M, Häyrynen T, Tulkki J, Oksanen J (2017). Generalized noise terms for the quantized fluctuational electrodynamics. J. Phys. B.

[16] Matloob R, Loudon R, Barnett SM, Jeffers J (1995). Electromagnetic field quantization in absorbing dielectrics. Phys. Rev. A.

[17] Matloob R, Loudon R (1996). Electromagnetic field quantization in absorbing dielectrics. II. Phys. Rev. A.

[18] Dung HT (2003). Electromagnetic-field quantization and spontaneous decay in left-handed media. Phys. Rev. A.

[19] Häyrynen T, Oksanen J (2016). Quantum description of light propagation in generalized media. J. Opt..

[20] Inoue K (2016). Quantum mechanical treatment of traveling light in an absorptive medium of two-level systems. Opt. Commun..

[21] Inoue K (2017). Quantum mechanical treatment of parametric amplification in an absorptive nonlinear medium. Opt. Commun..

[22] Roy Bardhan B, Shapiro JH (2016). Ultimate capacity of a linear time-invariant bosonic channel. Phys. Rev. A.

