# Supplementary material for: Interference-exact radiative transfer equation
Source: Sci Rep. 2017 Sep 14;7:11534. doi: 10.1038/s41598-017-11753-5 (PMC5599544; doi:10.1038/s41598-017-11753-5)
Supplement: Supplementary file 1 — Supplementary Information [file 41598_2017_11753_MOESM1_ESM.pdf]

# Supplemental Material for
# Interference-exact radiative transfer equation

Mikko Partanen,[1] Teppo Häyrynen,[2] and Jani Oksanen[1]

[1]*Engineered Nanosystems group, School of Science, Aalto University, P.O. Box 12200, 00076 Aalto, Finland*

[2]*DTU Fotonik, Department of Photonics Engineering, Technical University of Denmark, Ørsteds Plads, Building 343, DK-2800 Kongens Lyngby, Denmark*

(Dated: June 15, 2017)

## CONTENTS

I. Densities of states

II. Explicit forms of the RTE coefficients

III. Green's function components

IV. Homogeneous space
 A. Green's functions
 B. RTE coefficients

V. Single interface geometry
 A. Green's functions for normal incidence
 B. RTE coefficients for normal incidence

VI. Photon number transmission matrix

References

## I. DENSITIES OF STATES

Here we present the NLDOSs, IFDOSs, and LDOSs for the TE and TM polarizations of stratified media in terms of the spectral dyadic Green's function components $g_{jk}^{\alpha\beta}$, $i,j \in \{\mathrm{e},\mathrm{m}\}$, $\alpha,\beta \in \{1,2,3\}$. The NLDOSs are given as a function of the field and source point positions $z$ and $z'$, angular frequency $\omega$, and the wave vector component $K$ in the $x$-$y$ plane by [1]

$$\begin{aligned}\rho_{\mathrm{NL,TE}}(z,K,\omega,z') = \frac{\omega^3}{4\pi^3c^4}\Big[&|\varepsilon|\Big(\varepsilon_{\mathrm{i}}'|g_{\mathrm{ee}}^{11}|^2+\mu_{\mathrm{i}}'|g_{\mathrm{em}}^{12}|^2+\mu_{\mathrm{i}}'|g_{\mathrm{em}}^{13}|^2\Big)\\ &+|\mu|\Big(\varepsilon_{\mathrm{i}}'|g_{\mathrm{me}}^{21}|^2+\mu_{\mathrm{i}}'|g_{\mathrm{mm}}^{22}|^2+\mu_{\mathrm{i}}'|g_{\mathrm{mm}}^{23}|^2\\ &+\varepsilon_{\mathrm{i}}'|g_{\mathrm{me}}^{31}|^2+\mu_{\mathrm{i}}'|g_{\mathrm{mm}}^{32}|^2+\mu_{\mathrm{i}}'|g_{\mathrm{mm}}^{33}|^2\Big)\Big],\end{aligned} \tag{S1}$$

$$\begin{aligned}\rho_{\mathrm{NL,TM}}(z,K,\omega,z') = \frac{\omega^3}{4\pi^3c^4}\Big[&|\mu|\Big(\mu_{\mathrm{i}}'|g_{\mathrm{mm}}^{11}|^2+\varepsilon_{\mathrm{i}}'|g_{\mathrm{me}}^{12}|^2+\varepsilon_{\mathrm{i}}'|g_{\mathrm{me}}^{13}|^2\Big)\\ &+|\varepsilon|\Big(\mu_{\mathrm{i}}'|g_{\mathrm{em}}^{21}|^2+\varepsilon_{\mathrm{i}}'|g_{\mathrm{ee}}^{22}|^2+\varepsilon_{\mathrm{i}}'|g_{\mathrm{ee}}^{23}|^2\\ &+\mu_{\mathrm{i}}'|g_{\mathrm{em}}^{31}|^2+\varepsilon_{\mathrm{i}}'|g_{\mathrm{ee}}^{32}|^2+\varepsilon_{\mathrm{i}}'|g_{\mathrm{ee}}^{33}|^2\Big)\Big],\end{aligned} \tag{S2}$$

and the IFDOSs are given by [1]

$$\rho_{\mathrm{IF,TE}}(z,K,\omega,z') = -\frac{\omega^3 n_{\mathrm{r}}}{2\pi^3c^4}\mathrm{Im}\Big[\varepsilon_{\mathrm{i}}'g_{\mathrm{ee}}^{11}g_{\mathrm{me}}^{21*}+\mu_{\mathrm{i}}'g_{\mathrm{mm}}^{22}g_{\mathrm{em}}^{12*}+\mu_{\mathrm{i}}'g_{\mathrm{mm}}^{23}g_{\mathrm{em}}^{13*}\Big], \tag{S3}$$

$$\rho_{\text{IF,TM}}(z,K,\omega,z') = \frac{\omega^3 n_{\text{r}}}{2\pi^3 c^4}\text{Im}\Big[\mu_{\text{i}}' g_{\text{mm}}^{11} g_{\text{em}}^{21*} + \varepsilon_{\text{i}}' g_{\text{ee}}^{22} g_{\text{me}}^{12*} + \varepsilon_{\text{i}}' g_{\text{ee}}^{23} g_{\text{me}}^{13*}\Big]. \tag{S4}$$

Here $c$ is the speed of light in vacuum and $\varepsilon$ and $\mu$ are the relative permittivity and permeability of the medium written in terms of the real and imaginary parts as $\varepsilon = \varepsilon_{\text{r}} + i\varepsilon_{\text{i}}$ and $\mu = \mu_{\text{r}} + i\mu_{\text{i}}$. The refractive index of the medium is $n = \sqrt{\varepsilon\mu}$ and $n_{\text{r}}$ is its real part.

The LDOSs are integrals of the NLDOSs in Eqs. (S1) and (S2) with respect to $z'$. These integrals can be calculated analytically by using the Green's function identities. In terms of the imaginary parts of the Green's functions, the LDOSs for the TE and TM polarizations are then given by [1]

$$\rho_{\text{TE}}(z,K,\omega) = \frac{\omega}{4\pi^3 c^2}\text{Im}\Big[|\varepsilon| g_{\text{ee}}^{11} + |\mu| g_{\text{mm}}^{22} + \frac{\mu^2}{|\mu|} g_{\text{mm}}^{33}\Big], \tag{S5}$$

$$\rho_{\text{TM}}(z,K,\omega) = \frac{\omega}{4\pi^3 c^2}\text{Im}\Big[|\mu| g_{\text{mm}}^{11} + |\varepsilon| g_{\text{ee}}^{22} + \frac{\varepsilon^2}{|\varepsilon|} g_{\text{ee}}^{33}\Big]. \tag{S6}$$

## II. EXPLICIT FORMS OF THE RTE COEFFICIENTS

To obtain equations for the damping and scattering coefficients of the RTE model directly in terms of the Green's functions, we first need to calculate the derivatives of $\rho_{\text{NL},\sigma}$, $\rho_{\text{IF},\sigma}$, and $\rho_\sigma$. Assuming that material parameters are constant at position $z$, straightforward calculations using Eqs. (S1)–(S6) and Green's function identities give

$$\frac{\partial \rho_{\text{TE}}}{\partial z} = -\frac{\omega^2}{4\pi^3 c^3}\text{Im}\Big[\Big(|\varepsilon|\mu - \frac{|\mu| k_{\text{z}}^2}{k_0^2 \mu} + \frac{\mu K^2}{k_0^2|\mu|}\Big)\Big(g_{\text{em}}^{12} - g_{\text{me}}^{21}\Big)\Big], \tag{S7}$$

$$\frac{\partial \rho_{\text{TM}}}{\partial z} = \frac{\omega^2}{4\pi^3 c^3}\text{Im}\Big[\Big(|\mu|\varepsilon - \frac{|\varepsilon| k_{\text{z}}^2}{k_0^2 \varepsilon} + \frac{\varepsilon K^2}{k_0^2|\varepsilon|}\Big)\Big(g_{\text{me}}^{12} - g_{\text{em}}^{21}\Big)\Big], \tag{S8}$$

$$\begin{aligned}\frac{\partial \rho_{\text{NL,TE}}}{\partial z} = &\frac{\omega^4}{2\pi^3 c^5}\text{Re}\Big[\Big(|\varepsilon|\mu^* - \frac{k_{\text{z}}^2 - K^2}{k^2}|\mu|\varepsilon\Big)\\ &\times\Big(\varepsilon_{\text{i}}' g_{\text{ee}}^{11} g_{\text{me}}^{21*} - \mu_{\text{i}}' g_{\text{em}}^{12} g_{\text{mm}}^{22*} - \mu_{\text{i}}' g_{\text{em}}^{13} g_{\text{mm}}^{23*}\Big)\Big]\\ &+\frac{\omega^2}{4\pi^3 c^3}(|\mu|\varepsilon_{\text{i}} - |\varepsilon|\mu_{\text{i}})\text{Re}[g_{\text{em}}^{12} - g_{\text{me}}^{21}]\delta(z-z'),\end{aligned} \tag{S9}$$

$$\begin{aligned}\frac{\partial \rho_{\text{NL,TM}}}{\partial z} = &-\frac{\omega^4}{2\pi^3 c^5}\text{Re}\Big[\Big(|\mu|\varepsilon^* - \frac{k_{\text{z}}^2 - K^2}{k^2}|\varepsilon|\mu\Big)\\ &\times\Big(\mu_{\text{i}}' g_{\text{mm}}^{11} g_{\text{em}}^{21*} - \varepsilon_{\text{i}}' g_{\text{me}}^{12} g_{\text{ee}}^{22*} - \varepsilon_{\text{i}}' g_{\text{me}}^{13} g_{\text{ee}}^{23*}\Big)\Big]\\ &-\frac{\omega^2}{4\pi^3 c^3}(|\varepsilon|\mu_{\text{i}} - |\mu|\varepsilon_{\text{i}})\text{Re}[g_{\text{me}}^{12} - g_{\text{em}}^{21}]\delta(z-z'),\end{aligned} \tag{S10}$$

$$\begin{aligned}\frac{\partial \rho_{\text{IF,TE}}}{\partial z} = &-\frac{\omega^4 n_{\text{r}}}{2\pi^3 c^5}\Big[\text{Im}\Big[\frac{\varepsilon k_{\text{z}}^2}{k^2}\Big]\Big(\varepsilon_{\text{i}}'|g_{\text{ee}}^{11}|^2 + \mu_{\text{i}}'|g_{\text{em}}^{12}|^2 + \mu_{\text{i}}'|g_{\text{em}}^{13}|^2\Big)\\ &+\mu_{\text{i}}\Big(\varepsilon_{\text{i}}'|g_{\text{me}}^{21}|^2 + \mu_{\text{i}}'|g_{\text{mm}}^{22}|^2 + \mu_{\text{i}}'|g_{\text{mm}}^{23}|^2\Big)\Big]\\ &+\frac{\omega^2 n_{\text{r}}}{2\pi^3 c^3}\text{Im}\Big[\varepsilon_{\text{i}} g_{\text{ee}}^{11} + \mu_{\text{i}} g_{\text{mm}}^{22} + \mu_{\text{i}}\frac{\mu^2}{|\mu|^2} g_{\text{mm}}^{33}\Big]\delta(z-z'),\end{aligned} \tag{S11}$$

$$\begin{aligned}\frac{\partial \rho_{\text{IF,TM}}}{\partial z} = &-\frac{\omega^4 n_{\text{r}}}{2\pi^3 c^5}\Big[\text{Im}\Big[\frac{\mu k_{\text{z}}^2}{k^2}\Big]\Big(\mu_{\text{i}}'|g_{\text{mm}}^{11}|^2 + \varepsilon_{\text{i}}'|g_{\text{me}}^{12}|^2 + \varepsilon_{\text{i}}'|g_{\text{me}}^{13}|^2\Big)\\ &+\varepsilon_{\text{i}}\Big(\mu_{\text{i}}'|g_{\text{em}}^{21}|^2 + \varepsilon_{\text{i}}'|g_{\text{ee}}^{22}|^2 + \varepsilon_{\text{i}}'|g_{\text{ee}}^{23}|^2\Big)\Big]\\ &+\frac{\omega^2 n_{\text{r}}}{2\pi^3 c^3}\text{Im}\Big[\mu_{\text{i}} g_{\text{mm}}^{11} + \varepsilon_{\text{i}} g_{\text{ee}}^{22} + \varepsilon_{\text{i}}\frac{\varepsilon^2}{|\varepsilon|^2} g_{\text{ee}}^{33}\Big]\delta(z-z').\end{aligned} \tag{S12}$$

The second terms of Eqs. (S9)–(S12) are the delta function terms that contribute in the last terms of the damping and scattering coefficients in Eq. (2) of the manuscript. After substituting the Green's function expressions for the densities of states $\rho_\sigma$, $\rho_{\mathrm{NL},\sigma}$, and $\rho_{\mathrm{IF},\sigma}$ and their derivatives into Eq. (2) of the manuscript and simplifying the result, we obtain the damping and scattering coefficients as

$$\alpha_{\pm,\mathrm{TE}} = \frac{k_\mathrm{r} k_0}{\rho_\mathrm{TE}/\rho_0}\mathrm{Im}\Big[(\zeta_{\mathrm{e,TE}}+\varepsilon_\mathrm{i})g_\mathrm{ee}^{11}+(\zeta_{\mathrm{m,TE}}+\mu_\mathrm{i})g_\mathrm{mm}^{22}\pm\zeta_{\mathrm{ex,TE}}(g_\mathrm{me}^{21}-g_\mathrm{em}^{12})+\mu_\mathrm{i}\frac{\mu^2}{|\mu|^2}g_\mathrm{mm}^{33}\Big], \tag{S13}$$

$$\beta_{\pm,\mathrm{TE}} = \frac{k_\mathrm{r} k_0}{\rho_\mathrm{TE}/\rho_0}\mathrm{Im}\Big[(\zeta_{\mathrm{e,TE}}-\varepsilon_\mathrm{i})g_\mathrm{ee}^{11}+(\zeta_{\mathrm{m,TE}}-\mu_\mathrm{i})g_\mathrm{mm}^{22}\pm\zeta_{\mathrm{ex,TE}}^*(g_\mathrm{me}^{21}-g_\mathrm{em}^{12})-\mu_\mathrm{i}\frac{\mu^2}{|\mu|^2}g_\mathrm{mm}^{33}\Big], \tag{S14}$$

$$\alpha_{\pm,\mathrm{TM}} = \frac{k_\mathrm{r} k_0}{\rho_\mathrm{TM}/\rho_0}\mathrm{Im}\Big[(\zeta_{\mathrm{m,TM}}+\mu_\mathrm{i})g_\mathrm{mm}^{11}+(\zeta_{\mathrm{e,TM}}+\varepsilon_\mathrm{i})g_\mathrm{ee}^{22}\pm\zeta_{\mathrm{ex,TM}}(g_\mathrm{em}^{21}-g_\mathrm{me}^{12})+\varepsilon_\mathrm{i}\frac{\varepsilon^2}{|\varepsilon|^2}g_\mathrm{ee}^{33}\Big], \tag{S15}$$

$$\beta_{\pm,\mathrm{TM}} = \frac{k_\mathrm{r} k_0}{\rho_\mathrm{TM}/\rho_0}\mathrm{Im}\Big[(\zeta_{\mathrm{m,TM}}-\mu_\mathrm{i})g_\mathrm{mm}^{11}+(\zeta_{\mathrm{e,TM}}-\varepsilon_\mathrm{i})g_\mathrm{ee}^{22}\pm\zeta_{\mathrm{ex,TM}}^*(g_\mathrm{em}^{21}-g_\mathrm{me}^{12})-\varepsilon_\mathrm{i}\frac{\varepsilon^2}{|\varepsilon|^2}g_\mathrm{ee}^{33}\Big], \tag{S16}$$

where $k_\mathrm{r}$ is the real part of the wavenumber in the medium, $\rho_0 = 1/(4\pi^3 c)$ is the LDOS for a single polarization in vacuum for $K = 0$, and the complex coefficients $\zeta_{\mathrm{e},\sigma}$, $\zeta_{\mathrm{m},\sigma}$, and $\zeta_{\mathrm{ex},\sigma}$ are given for the TE and TM polarizations by

$$\begin{aligned}
\zeta_{\mathrm{e,TE}} &= 2i\varepsilon+\zeta_\mathrm{c}\frac{k_z}{k_0\mu}, & \zeta_{\mathrm{e,TM}} &= 2i\varepsilon+\zeta_\mathrm{c}\frac{k_0\varepsilon}{k_z},\\
\zeta_{\mathrm{m,TE}} &= 2i\mu+\zeta_\mathrm{c}\frac{k_0\mu}{k_z}, & \zeta_{\mathrm{m,TM}} &= 2i\mu+\zeta_\mathrm{c}\frac{k_z}{k_0\varepsilon},\\
\zeta_{\mathrm{ex,TE}} &= \frac{1}{2n_\mathrm{r}}\Big(|\varepsilon|\mu-|\mu|\varepsilon+\frac{2\mu_\mathrm{r}K^2}{k_0^2|\mu|}\Big), & \zeta_{\mathrm{ex,TM}} &= -\frac{1}{2n_\mathrm{r}}\Big(|\mu|\varepsilon-|\varepsilon|\mu+\frac{2\varepsilon_\mathrm{r}K^2}{k_0^2|\varepsilon|}\Big).
\end{aligned} \tag{S17}$$

Here the parameter $\zeta_\mathrm{c}$ is given by

$$\zeta_\mathrm{c} = \mathrm{Im}\Big[\frac{k_z(|k|^2+|k_z|^2+K^2)^2}{4k_0k_\mathrm{r}^2|k_z|^2}+\frac{k^2+k_z^2}{k_0k_z}\Big]+i\mathrm{Re}\Big[\frac{k_z(|k|^2-|k_z|^2+K^2)^2}{4k_0k_\mathrm{r}^2|k_z|^2}-\frac{k^2+k_z^2}{k_0k_z}\Big] \tag{S18}$$

As the real parts of the exchange Green's functions $g_\mathrm{me}^{21}(z,K,\omega,z')$ and $g_\mathrm{em}^{12}(z,K,\omega,z')$ are discontinuous at $z=z'$, we have defined $g_\mathrm{me}^{21}-g_\mathrm{em}^{12}$ in Eqs. (S13)–(S16) as a limit $g_\mathrm{me}^{21}-g_\mathrm{em}^{12} = \lim_{z'\to z}[g_\mathrm{me}^{21}(z,K,\omega,z')-g_\mathrm{em}^{12}(z,K,\omega,z')]$, which is well-defined despite the discontinuity since the discontinuity cancels out.

## III. GREEN'S FUNCTION COMPONENTS

Here we present the components of the spectral dyadic Green's functions $g_\mathrm{ee}^{\alpha\beta}(z,K,\omega,z')$ of stratified media in terms of the scalar Green's function of the TE polarization for electric sources denoted by $\xi_{\mathrm{e},\|}(z,\omega,z')$ and the scalar Green's function of TM polarization for magnetic sources denoted by $\xi_{\mathrm{m},\|}(z,\omega,z')$. Explicit forms for the scalar Green's functions $\xi_{\mathrm{e},\|}(z,\omega,z')$ and $\xi_{\mathrm{m},\|}(z,\omega,z')$ can be found, e.g., in Ref. [1] or, alternatively, they can be calculated, e.g., by using the techniques presented in Refs. [2] or [3]. The resulting relations for the electric spectral Green's function components $g_\mathrm{ee}^{\alpha\beta}(z,K,\omega,z')$ are then given by

$$g_\mathrm{ee}^{11}(z,K,\omega,z') = \mu'\xi_{\mathrm{e},\|}(z,\omega,z'), \tag{S19}$$

$$g_\mathrm{ee}^{22}(z,K,\omega,z') = \frac{\mu'}{k'^2}\frac{\partial^2}{\partial z\partial z'}\xi_{\mathrm{m},\|}(z',\omega,z)-\frac{\mu'}{k'^2}\delta(z-z'), \tag{S20}$$

$$g_\mathrm{ee}^{23}(z,K,\omega,z') = \mu'\frac{iK}{k'^2}\frac{\partial}{\partial z}\xi_{\mathrm{m},\|}(z',\omega,z), \tag{S21}$$

$$g_{\rm ee}^{32}(z,K,\omega,z') = -\mu' \frac{iK}{k'^2}\frac{\partial}{\partial z'}\xi_{\rm m,\parallel}(z',\omega,z), \tag{S22}$$

$$g_{\rm ee}^{33}(z,K,\omega,z') = \mu' \frac{K^2}{k'^2}\xi_{\rm m,\parallel}(z',\omega,z) - \frac{\mu'}{k'^2}\delta(z-z'). \tag{S23}$$

The Dirac delta function in the second term of Eq. (S20) is cancelled by a delta function resulting from the derivatives in the first term. The delta function in the second term of Eq. (S23) is, however, not cancelled, but it is neglected as the value of $g_{\rm ee}^{33}(z,K,\omega,z')$ at $z'=z$ is determined in the limit $z' \longrightarrow z$. The corresponding relations for the magnetic spectral Green's function components $g_{\rm mm}^{\alpha\beta}(z,K,\omega,z')$ are given by

$$g_{\rm mm}^{11}(z,K,\omega,z') = \varepsilon' \xi_{\rm m,\parallel}(z,\omega,z'), \tag{S24}$$

$$g_{\rm mm}^{22}(z,K,\omega,z') = \frac{\varepsilon'}{k'^2}\frac{\partial^2}{\partial z \partial z'}\xi_{\rm e,\parallel}(z',\omega,z) - \frac{\varepsilon'}{k'^2}\delta(z-z'), \tag{S25}$$

$$g_{\rm mm}^{23}(z,K,\omega,z') = \varepsilon' \frac{iK}{k'^2}\frac{\partial}{\partial z}\xi_{\rm e,\parallel}(z',\omega,z), \tag{S26}$$

$$g_{\rm mm}^{32}(z,K,\omega,z') = -\varepsilon' \frac{iK}{k'^2}\frac{\partial}{\partial z'}\xi_{\rm e,\parallel}(z',\omega,z), \tag{S27}$$

$$g_{\rm mm}^{33}(z,K,\omega,z') = \varepsilon' \frac{K^2}{k'^2}\xi_{\rm e,\parallel}(z',\omega,z) - \frac{\varepsilon'}{k'^2}\delta(z-z'). \tag{S28}$$

The delta function relations discussed above in the case of $g_{\rm ee}^{\alpha\beta}(z,K,\omega,z')$ also apply to $g_{\rm mm}^{\alpha\beta}(z,K,\omega,z')$. The relations for the spectral exchange Green's function components $g_{\rm me}^{\alpha\beta}(z,K,\omega,z')$ are given by

$$g_{\rm me}^{12}(z,K,\omega,z') = \frac{\mu'}{\mu}\frac{k^2}{k'^2}\frac{\partial}{k_0 \partial z'}\xi_{\rm m,\parallel}(z',\omega,z), \tag{S29}$$

$$g_{\rm me}^{13}(z,K,\omega,z') = \frac{\mu'}{\mu}\frac{iKk^2}{k_0 k'^2}\xi_{\rm m,\parallel}(z',\omega,z), \tag{S30}$$

$$g_{\rm me}^{21}(z,K,\omega,z') = \frac{\mu'}{\mu}\frac{\partial}{k_0 \partial z}\xi_{\rm e,\parallel}(z,\omega,z'), \tag{S31}$$

$$g_{\rm me}^{31}(z,K,\omega,z') = -\frac{\mu'}{\mu}\frac{iK}{k_0}\xi_{\rm e,\parallel}(z,\omega,z'), \tag{S32}$$

and the relations for the spectral exchange Green's function components $g_{\rm em}^{\alpha\beta}(z,K,\omega,z')$ are, respectively, given by

$$g_{\rm em}^{12}(z,K,\omega,z') = -\frac{\varepsilon'}{\varepsilon}\frac{k^2}{k'^2}\frac{\partial}{k_0 \partial z'}\xi_{\rm e,\parallel}(z',\omega,z), \tag{S33}$$

$$g_{\rm em}^{13}(z,K,\omega,z') = -\frac{\varepsilon'}{\varepsilon}\frac{iKk^2}{k_0 k'^2}\xi_{\rm e,\parallel}(z',\omega,z), \tag{S34}$$

$$g_{\rm em}^{21}(z,K,\omega,z') = -\frac{\varepsilon'}{\varepsilon}\frac{\partial}{k_0 \partial z}\xi_{\rm m,\parallel}(z,\omega,z'), \tag{S35}$$

$$g_{\rm em}^{31}(z,K,\omega,z') = \frac{\varepsilon'}{\varepsilon}\frac{iK}{k_0}\xi_{\rm m,\parallel}(z,\omega,z'). \tag{S36}$$

## IV. HOMOGENEOUS SPACE

### A. Green's functions

In a homogeneous space, the auxiliary Green's functions are given by $\xi_{\mathrm{e},\|}(z,\omega,z') = \xi_{\mathrm{m},\|}(z,\omega,z') = ie^{ik_z|z-z'|}/(2k_z)$ and, using Eqs. (S19)–(S23), the relations for the electric spectral Green's function components $g_{\mathrm{ee}}^{\alpha\beta}(z,K,\omega,z')$ are given by

$$g_{\mathrm{ee}}^{11}(z,K,\omega,z') = \frac{i\mu}{2k_z}e^{ik_z|z-z'|}, \tag{S37}$$

$$g_{\mathrm{ee}}^{22}(z,K,\omega,z') = \frac{i\mu k_z}{2k^2}e^{ik_z|z-z'|}, \tag{S38}$$

$$g_{\mathrm{ee}}^{23}(z,K,\omega,z') = -\frac{i\mu K}{2k^2}\Big(\theta(z-z')e^{ik_z(z-z')} - \theta(z'-z)e^{-ik_z(z-z')}\Big), \tag{S39}$$

$$g_{\mathrm{ee}}^{32}(z,K,\omega,z') = -\frac{i\mu K}{2k^2}\Big(\theta(z-z')e^{ik_z(z-z')} - \theta(z'-z)e^{-ik_z(z-z')}\Big), \tag{S40}$$

$$g_{\mathrm{ee}}^{33}(z,K,\omega,z') = \frac{i\mu K^2}{2k^2k_z}e^{ik_z|z-z'|} - \frac{\mu}{k^2}\delta(z-z'). \tag{S41}$$

The corresponding relations for the magnetic spectral Green's function components $g_{\mathrm{mm}}^{\alpha\beta}(z,K,\omega,z')$, following from Eqs. (S24)–(S28), are given by

$$g_{\mathrm{mm}}^{11}(z,K,\omega,z') = \frac{i\varepsilon}{2k_z}e^{ik_z|z-z'|}, \tag{S42}$$

$$g_{\mathrm{mm}}^{22}(z,K,\omega,z') = \frac{i\varepsilon k_z}{2k^2}e^{ik_z|z-z'|}, \tag{S43}$$

$$g_{\mathrm{mm}}^{23}(z,K,\omega,z') = -\frac{i\varepsilon K}{2k^2}\Big(\theta(z-z')e^{ik_z(z-z')} - \theta(z'-z)e^{-ik_z(z-z')}\Big), \tag{S44}$$

$$g_{\mathrm{mm}}^{32}(z,K,\omega,z') = -\frac{i\varepsilon K}{2k^2}\Big(\theta(z-z')e^{ik_z(z-z')} - \theta(z'-z)e^{-ik_z(z-z')}\Big), \tag{S45}$$

$$g_{\mathrm{mm}}^{33}(z,K,\omega,z') = \frac{i\varepsilon K^2}{2k^2k_z}e^{ik_z|z-z'|} - \frac{\varepsilon}{k^2}\delta(z-z'). \tag{S46}$$

The relations for the spectral exchange Green's function components $g_{\mathrm{me}}^{\alpha\beta}(z,K,\omega,z')$, following from Eqs. (S29)–(S32), are given by

$$g_{\mathrm{me}}^{12}(z,K,\omega,z') = \frac{1}{2k_0}\Big(\theta(z-z')e^{ik_z(z-z')} - \theta(z'-z)e^{-ik_z(z-z')}\Big), \tag{S47}$$

$$g_{\mathrm{me}}^{13}(z,K,\omega,z') = -\frac{K}{2k_0k_z}e^{ik_z|z-z'|}, \tag{S48}$$

$$g_{\mathrm{me}}^{21}(z,K,\omega,z') = -\frac{1}{2k_0}\Big(\theta(z-z')e^{ik_z(z-z')} - \theta(z'-z)e^{-ik_z(z-z')}\Big), \tag{S49}$$

$$g_{\rm me}^{31}(z,K,\omega,z') = \frac{K}{2k_0 k_z} e^{ik_z|z-z'|}, \tag{S50}$$

and, using Eqs. (S33)–(S36), the relations for the spectral exchange Green's function components $g_{\rm em}^{\alpha\beta}(z,K,\omega,z')$ are, respectively, given by

$$g_{\rm em}^{12}(z,K,\omega,z') = -\frac{1}{2k_0}\Big(\theta(z-z')e^{ik_z(z-z')} - \theta(z'-z)e^{-ik_z(z-z')}\Big), \tag{S51}$$

$$g_{\rm em}^{13}(z,K,\omega,z') = \frac{K}{2k_0 k_z} e^{ik_z|z-z'|}, \tag{S52}$$

$$g_{\rm em}^{21}(z,K,\omega,z') = \frac{1}{2k_0}\Big(\theta(z-z')e^{ik_z(z-z')} - \theta(z'-z)e^{-ik_z(z-z')}\Big), \tag{S53}$$

$$g_{\rm em}^{31}(z,K,\omega,z') = -\frac{K}{2k_0 k_z} e^{ik_z|z-z'|}. \tag{S54}$$

### B. RTE coefficients

Substituting the homogeneous space Green's function components from Eqs. (S37)–(S54) into the formulas of the RTE coefficients in terms of the Green's function components in Eqs. (S13)–(S16) gives

$$\alpha_{\pm,\rm TE} = k_{\rm r}\frac{\mathrm{Re}\Big[(\zeta_{\rm e,TE}+\varepsilon_{\rm i})\frac{\mu}{2k_z} + (\zeta_{\rm m,TE}+\mu_{\rm i})\frac{\varepsilon k_z}{2k^2} + \mu_{\rm i}\frac{\mu^2}{|\mu|^2}\frac{\varepsilon K^2}{2k^2k_z}\Big]}{\mathrm{Re}\Big[|\varepsilon|\frac{\mu}{2k_z} + |\mu|\frac{\varepsilon k_z}{2k^2} + \frac{\mu^2}{|\mu|}\frac{\varepsilon K^2}{2k^2k_z}\Big]}, \tag{S55}$$

$$\beta_{\pm,\rm TE} = k_{\rm r}\frac{\mathrm{Re}\Big[(\zeta_{\rm e,TE}-\varepsilon_{\rm i})\frac{\mu}{2k_z} + (\zeta_{\rm m,TE}-\mu_{\rm i})\frac{\varepsilon k_z}{2k^2} - \mu_{\rm i}\frac{\mu^2}{|\mu|^2}\frac{\varepsilon K^2}{2k^2k_z}\Big]}{\mathrm{Re}\Big[|\varepsilon|\frac{\mu}{2k_z} + |\mu|\frac{\varepsilon k_z}{2k^2} + \frac{\mu^2}{|\mu|}\frac{\varepsilon K^2}{2k^2k_z}\Big]}, \tag{S56}$$

$$\alpha_{\pm,\rm TM} = k_{\rm r}\frac{\mathrm{Re}\Big[(\zeta_{\rm m,TM}+\mu_{\rm i})\frac{\varepsilon}{2k_z} + (\zeta_{\rm e,TM}+\varepsilon_{\rm i})\frac{\mu k_z}{2k^2} + \varepsilon_{\rm i}\frac{\varepsilon^2}{|\varepsilon|^2}\frac{\mu K^2}{2k^2k_z}\Big]}{\mathrm{Re}\Big[|\mu|\frac{\varepsilon}{2k_z} + |\varepsilon|\frac{\mu k_z}{2k^2} + \frac{\varepsilon^2}{|\varepsilon|}\frac{\mu K^2}{2k^2k_z}\Big]}, \tag{S57}$$

$$\beta_{\pm,\rm TM} = k_{\rm r}\frac{\mathrm{Re}\Big[(\zeta_{\rm m,TM}-\mu_{\rm i})\frac{\varepsilon}{2k_z} + (\zeta_{\rm e,TM}-\varepsilon_{\rm i})\frac{\mu k_z}{2k^2} - \varepsilon_{\rm i}\frac{\varepsilon^2}{|\varepsilon|^2}\frac{\mu K^2}{2k^2k_z}\Big]}{\mathrm{Re}\Big[|\mu|\frac{\varepsilon}{2k_z} + |\varepsilon|\frac{\mu k_z}{2k^2} + \frac{\varepsilon^2}{|\varepsilon|}\frac{\mu K^2}{2k^2k_z}\Big]}, \tag{S58}$$

Simplifying these equations further, gives the formulas presented in Eq. (3) of the manuscript.

## V. SINGLE INTERFACE GEOMETRY

### A. Green's functions for normal incidence

Here we present the spectral Green's functions for normal incidence, i.e., for $K=0$, in a single interface geometry. For normal incidence, the Green's functions for the TE and TM polarizations are equal. The single interface geometry is formed from two media with relative permittivities and permeabilities $\varepsilon_1$, $\mu_1$ ($z<0$) and $\varepsilon_2$, $\mu_2$ ($z>0$). The corresponding refractive indices are denoted by $n_1$ and $n_2$, and the wavenumbers are denoted by $k_1$ and $k_2$. The electric and magnetic Green's functions are given for normal incidence by [1]

$$g_{\rm ee}^{11}(z,\omega,z') = \mu(z',\omega)\xi_{\rm e}(z,\omega,z'), \tag{S59}$$
$$g_{\rm mm}^{22}(z,\omega,z') = \varepsilon(z',\omega)\xi_{\rm m}(z,\omega,z'), \tag{S60}$$

where the auxiliary Green's functions $\xi_j(z,\omega,z')$ are given for the single interface geometry in the left half-space ($z<0$) by [1]

$$\xi_{j,\{z<0\}}(z,\omega,z') = \frac{i\,\theta(-z')}{2k_1}\Big(e^{ik_1|z-z'|}+r_j e^{-ik_1(z+z')}\Big)+\frac{i\,\theta(z')}{2k_2}t'_j e^{-i(k_1x-k_2x')} \tag{S61}$$

and on the right half-space ($z>0$) by

$$\xi_{j,\{z>0\}}(z,\omega,z') = \frac{i\,\theta(-z')}{2k_1}t_j e^{i(k_2x-k_1x')}+\frac{i\,\theta(z')}{2k_2}\Big(e^{ik_2|z-z'|}+r'_j e^{ik_2(z+z')}\Big). \tag{S62}$$

Here, $\theta(z)$ is the step function and $r_\mathrm{e}$, $r_\mathrm{m}$, $t_\mathrm{e}$, and $t_\mathrm{m}$ are the Fresnel reflection and transmission coefficients for left normal incidence, given by

$$\begin{aligned} r_\mathrm{e} &= \frac{\mu_2 n_1-\mu_1 n_2}{\mu_2 n_1+\mu_1 n_2}, & t_\mathrm{e} &= \frac{2\mu_2 n_1}{\mu_2 n_1+\mu_1 n_2},\\ r_\mathrm{m} &= \frac{\varepsilon_2 n_1-\varepsilon_1 n_2}{\varepsilon_2 n_1+\varepsilon_1 n_2}, & t_\mathrm{m} &= \frac{2\varepsilon_2 n_1}{\varepsilon_2 n_1+\varepsilon_1 n_2}. \end{aligned} \tag{S63}$$

The reflection and transmission coefficients for right normal incidence $r'_\mathrm{e}$, $r'_\mathrm{m}$, $t'_\mathrm{e}$, and $t'_\mathrm{m}$ are obtained by switching the indices 1 and 2 in Eq. (S63). In the auxiliary Green's functions of both half-spaces in Eqs. (S61) and (S62), the first term describes the field generated by the source point in the left half-space, whereas the second term describes the field generated by the source source point in the right half-space.

### B. RTE coefficients for normal incidence

Substituting the normal incidence Green's functions of the single interface geometry given in Eqs. (S59) and (S60), the damping and scattering coefficients in Eqs. (S13)–(S16) become equal for the TE and TM polarizations and are given in the left medium as

$$\begin{aligned} \alpha_{1,\pm} &= k_{1,\mathrm{r}}\frac{\mathrm{Re}\Big[\zeta_+ + \Big(\frac{\varepsilon_{1,\mathrm{i}}\mu_1-\mu_{1,\mathrm{i}}\varepsilon_1}{n_1}\pm 2i\zeta_{\mathrm{ex},1}\Big)r_\mathrm{e}e^{-2ik_1x}\Big]}{\mathrm{Re}\Big[\frac{|\varepsilon_1|\mu_1+|\mu_1|\varepsilon_1}{n_1}+\frac{|\varepsilon_1|\mu_1-|\mu_1|\varepsilon_1}{n_1}r_\mathrm{e}e^{-2ik_1x}\Big]},\\ \beta_{1,\pm} &= k_{1,\mathrm{r}}\frac{\mathrm{Re}\Big[\zeta_- + \Big(\frac{\mu_{1,\mathrm{i}}\varepsilon_1-\varepsilon_{1,\mathrm{i}}\mu_1}{n_1}\pm 2i\zeta^*_{\mathrm{ex},1}\Big)r_\mathrm{e}e^{-2ik_1x}\Big]}{\mathrm{Re}\Big[\frac{|\varepsilon_1|\mu_1+|\mu_1|\varepsilon_1}{n_1}+\frac{|\varepsilon_1|\mu_1-|\mu_1|\varepsilon_1}{n_1}r_\mathrm{e}e^{-2ik_1x}\Big]}, \end{aligned} \tag{S64}$$

where the parameters $\zeta_+$ and $\zeta_-$ are given by

$$\zeta_\pm = \frac{2n_{1,\mathrm{i}}|n_1|^2}{n_{1,\mathrm{r}}^2}\pm\frac{\varepsilon_{1,\mathrm{i}}\mu_1+\mu_{1,\mathrm{i}}\varepsilon_1}{n_1}. \tag{S65}$$

Respectively, the damping and scattering coefficients are given in the right medium by

$$\begin{aligned} \alpha_{2,\pm} &= k_{2,\mathrm{r}}\frac{\mathrm{Re}\Big[\zeta_+ + \Big(\frac{\varepsilon_{2,\mathrm{i}}\mu_2-\mu_{2,\mathrm{i}}\varepsilon_2}{n_2}\mp 2i\zeta_{\mathrm{ex},2}\Big)r'_\mathrm{e}e^{2ik_2x}\Big]}{\mathrm{Re}\Big[\frac{|\varepsilon_2|\mu_2+|\mu_2|\varepsilon_2}{n_2}+\frac{|\varepsilon_2|\mu_2-|\mu_2|\varepsilon_2}{n_2}r'_\mathrm{e}e^{2ik_2x}\Big]},\\ \beta_{2,\pm} &= k_{2,\mathrm{r}}\frac{\mathrm{Re}\Big[\zeta_- + \Big(\frac{\mu_{2,\mathrm{i}}\varepsilon_2-\varepsilon_{2,\mathrm{i}}\mu_2}{n_2}\mp 2i\zeta^*_{\mathrm{ex},2}\Big)r'_\mathrm{e}e^{2ik_2x}\Big]}{\mathrm{Re}\Big[\frac{|\varepsilon_2|\mu_2+|\mu_2|\varepsilon_2}{n_2}+\frac{|\varepsilon_2|\mu_2-|\mu_2|\varepsilon_2}{n_2}r'_\mathrm{e}e^{2ik_2x}\Big]}. \end{aligned} \tag{S66}$$

Far from the interface, here the exponential terms become zero, the damping and scattering coefficients in Eq. (S64) simplify to the homogeneous space coefficients in Eq. (3) of the manuscript, in which we must additionally set $K=0$ for normal incidence.

## VI. PHOTON NUMBER TRANSMISSION MATRIX

At interfaces between homogeneous media, the material parameters and densities of states are generally discontinuous. To account for this one might model the material parameter discontinuities by using continuous but rapidly varying functions or include $\delta$-functions to the damping and scattering coefficients. A more straightforward approach is to apply the photon number transmission coefficients. The photon number transmission coefficients are incorporated in a photon number transmission matrix $\mathbf{T}_{\sigma,i}$, which relates the photon numbers on the left $(z = z_i^-)$ and right $(z = z_i^+)$ of an interface at position $z = z_i$ as $\mathbf{n}_\sigma(z_i^+) = \mathbf{T}_{\sigma,i}\mathbf{n}_\sigma(z_i^-)$. The photon number transmission matrix is written as

$$\mathbf{T}_{\sigma,i} = \begin{bmatrix} T_{\sigma,i}^{11} & T_{\sigma,i}^{12} \\ T_{\sigma,i}^{21} & T_{\sigma,i}^{22} \end{bmatrix}. \tag{S67}$$

The entries of this photon number transmission matrix are given by

$$\begin{aligned} T_{\sigma,i}^{11} &= \frac{t_{+,\sigma,i}t'_{-,\sigma,i} - r_{+,\sigma,i}r'_{-,\sigma,i}}{t'_{-,\sigma,i} - r_{+,\sigma,i}r'_{-,\sigma,i}t'_{+,\sigma,i}}, & T_{\sigma,i}^{12} &= \frac{r'_{-,\sigma,i}(1 - t_{+,\sigma,i}t'_{+,\sigma,i})}{t'_{-,\sigma,i} - r_{+,\sigma,i}r'_{-,\sigma,i}t'_{+,\sigma,i}}, \\ T_{\sigma,i}^{21} &= \frac{-r_{+,\sigma,i}(1 - t_{-,\sigma,i}t'_{-,\sigma,i})}{t'_{-,\sigma,i} - r_{+,\sigma,i}r'_{-,\sigma,i}t'_{+,\sigma,i}}, & T_{\sigma,i}^{22} &= \frac{1 - r_{+,\sigma,i}r'_{-,\sigma,i}t_{-,\sigma,i}t'_{+,\sigma,i}}{t'_{-,\sigma,i} - r_{+,\sigma,i}r'_{-,\sigma,i}t'_{+,\sigma,i}}, \end{aligned} \tag{S68}$$

where the quantities $r_{\pm,\sigma,i}$ and $t_{\pm,\sigma,i}$ correspond to the single interface photon number reflection and transmission coefficients for the left incidence. The primed quantities are the corresponding single interface coefficients for the right incidence. In terms of the NLDOSs, these coefficients are given by

$$\begin{aligned} r_{\pm,\sigma,i} &= \frac{\rho_{\mathrm{NL}\mp,\sigma}(z_i^-, z_i^{--})}{\rho_{\mathrm{NL}\pm,\sigma}(z_i^-, z_i^{--})}, & r'_{\pm,\sigma,i} &= \frac{\rho_{\mathrm{NL}\mp,\sigma}(z_i^+, z_i^{++})}{\rho_{\mathrm{NL}\pm,\sigma}(z_i^+, z_i^{++})}, \\ t_{\pm,\sigma,i} &= \frac{\rho_\sigma(z_i^-)}{\rho_\sigma(z_i^+)}\frac{\rho_{\mathrm{NL}\pm,\sigma}(z_i^+, z_i^{--})}{\rho_{\mathrm{NL}\pm,\sigma}(z_i^-, z_i^{--})}, & t'_{\pm,\sigma,i} &= \frac{\rho_\sigma(z_i^+)}{\rho_\sigma(z_i^-)}\frac{\rho_{\mathrm{NL}\pm,\sigma}(z_i^-, z_i^{++})}{\rho_{\mathrm{NL}\pm,\sigma}(z_i^+, z_i^{++})}, \end{aligned} \tag{S69}$$

where $z_i^{--}$ is any position coordinate on the left of $z = z_i^-$ while $z_i^{++}$ is any position coordinate on the right of $z = z_i^+$.


[1] M. Partanen, T. Häyrynen, J. Tulkki, and J. Oksanen, "Quantized fluctuational electrodynamics for three-dimensional plasmonic structures," *Phys. Rev. A* **95**, 013848 (2017).

[2] O. D. Stefano, S. Savasta, and R. Girlanda, "Mode expansion and photon operators in dispersive and absorbing dielectrics," *J. Mod. Opt.* **48**, 67 (2001).

[3] M. Paulus, P. Gay-Balmaz, and O. J. F. Martin, "Accurate and efficient computation of the green's tensor for stratified media," *Phys. Rev. E* **62**, 5797 (2000).
